# When the Missing Link Was a Drink: Missed Diagnosis of a Lung Mass Due to Limited History

**DOI:** 10.7759/cureus.76956

**Published:** 2025-01-05

**Authors:** Rugved Parmar, Sucharitha Pandeti, Linda Okoro, Gopi Venkatesvaran, Ahmed Kawamj, Michael J Akerman

**Affiliations:** 1 Internal Medicine, B. J. (Byramjee Jeejeebhoy) Medical College, Ahmedabad, IND; 2 Internal Medicine, Sri Venkateswara Medical College, Tirupati, IND; 3 Internal Medicine, New York Medical College, New York, USA; 4 Internal Medicine, St. Mary's Hospital, Passaic, USA; 5 Internal Medicine, Saint Clare’s Health, Denville, USA; 6 Internal Medicine/Pulmonary and Critical Care Medicine, New York Medical College, New York, USA; 7 Internal Medicine, Touro College of Osteopathic Medicine, New York, USA; 8 Internal Medicine/Pulmonary and Critical Care Medicine, St. Mary's Hospital, Passaic, USA; 9 Internal Medicine/Pulmonary and Critical Care Medicine, Saint Clare’s Health, Denville, USA

**Keywords:** alcohol use, art of diagnosis, aspiration, lung abscess, lung mass, medical history, patient centered, pulmonary aspergillosis, video-assisted thoracoscopic surgery, young adult male

## Abstract

A 24-year-old male patient presented with a two-month history of productive cough and hemoptysis. Chest imaging revealed a 3.2 cm cavitating lesion in the right lower lobe, initially suspected to be either an inflammatory mass, neoplasm, or aspergilloma. He underwent video-assisted thoracoscopy with a right lower lobectomy which revealed a 4.5 cm cavitary mass and lymphoid hyperplasia, consistent with aspiration lung abscess. Post-operatively, the patient experienced a persistent pneumothorax requiring extended monitoring and follow-up. Subsequently, the patient disclosed a history of recurrent binge drinking with episodes of unconsciousness, establishing the etiology as aspiration-related. The delayed identification of the alcohol use history contributed to a missed diagnosis of aspiration lung abscess. As a result, the patient was not treated with intravenous antibiotics, which might have obviated the need for a lobectomy and its attendant surgical risk. This report underscores the critical importance of allocating sufficient time and effort to obtain a thorough clinical history.

## Introduction

A lung abscess is a localized area of parenchymal necrosis caused by infection, resulting in the formation of one or more suppurative cavities. The presence of a bronchial fistula can lead to an air-fluid level visible on imaging [1]. Lung abscesses are classified as acute (lasting less than six weeks) or chronic (lasting more than six weeks). In adults, they are further categorized based on etiology: primary abscesses often arise from aspiration of oropharyngeal secretions or necrotizing pneumonitis, while secondary abscesses may develop due to bronchial obstruction, hematogenous spread, contiguous infection from the mediastinum, or other underlying lung diseases [2].

Before the antibiotic era, surgical intervention was the mainstay of treatment. Currently, however, most patients respond well to intravenous antibiotics, with surgery reserved as a treatment of last resort, due to its increased mortality risk, which can exceed 11% [3,4]. The patient interview remains a key diagnostic tool, utilizing the clinical history to guide the appropriate diagnostic and treatment interventions [5]. This case highlights diagnostic challenges and clinical management pitfalls of lung abscesses, particularly when significant risk factors remain unrecognized.

## Case presentation

A 24-year-old male patient was referred to our internal medicine resident continuity clinic by his primary care physician (PCP). He first sought care from his PCP two months earlier with the onset of a persistent cough. One month prior to his visit here, his cough became productive of off-color sputum. Several days prior to presentation, he developed small amounts of daily hemoptysis. An oral amoxicillin course from his PCP and self-treatment with over-the-counter medications had not provided relief.

**Figure 1:**
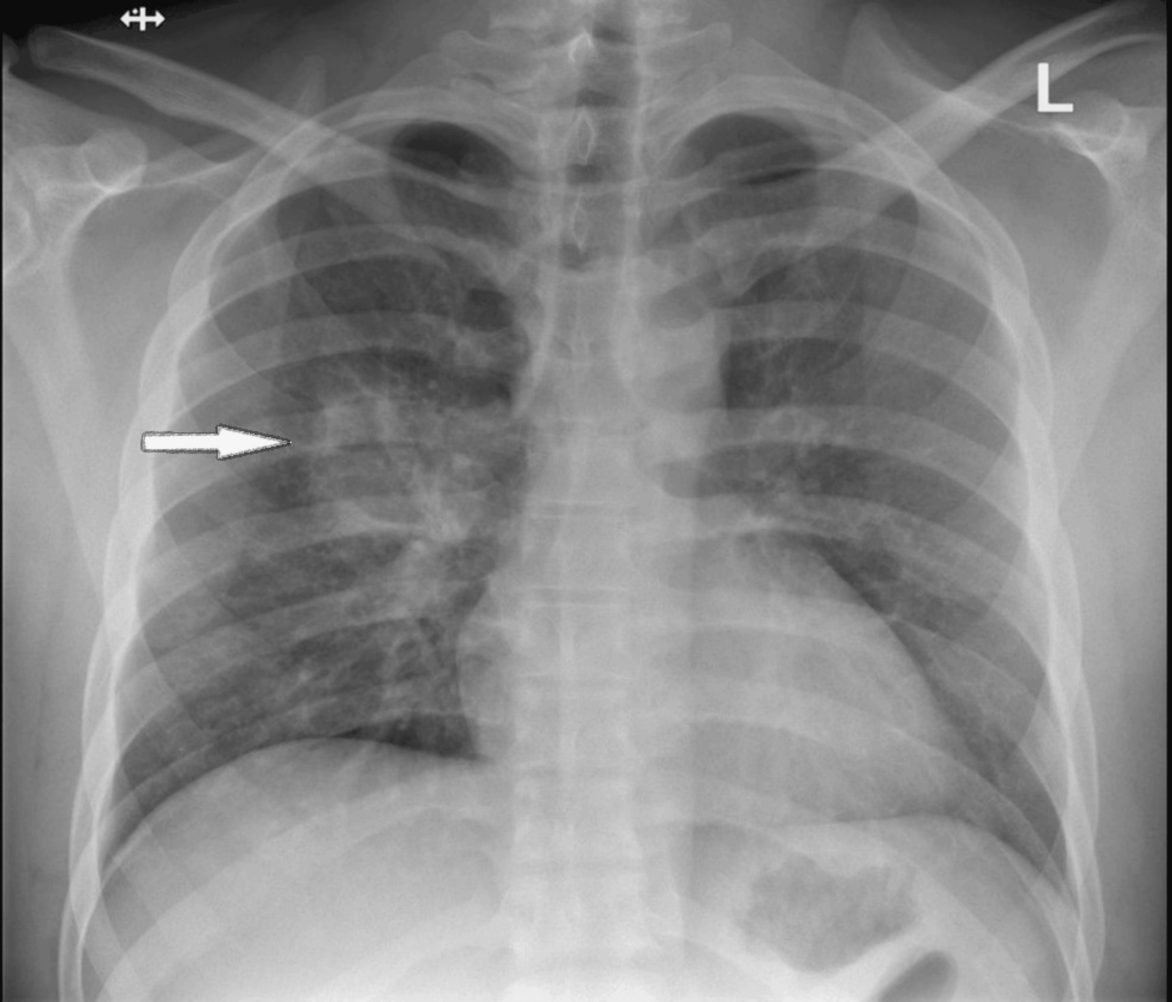
Chest X-ray showing a mass-like lung lesion located at the right hilum (arrow)

The patient was generally in good health with no significant past medical history. He denied fevers, chills, night sweats, or weight loss. There was no family history of malignancy. He worked with a sports team and traveled extensively across the entire United States. He denied any substance use or tobacco use. He reported “social” alcohol consumption, which he had discontinued since symptom onset. Six days prior to his visit to our clinic, his PCP had ordered a chest X-ray, which revealed a 5 cm right perihilar mass (Figure 1).

**Figure 2:**
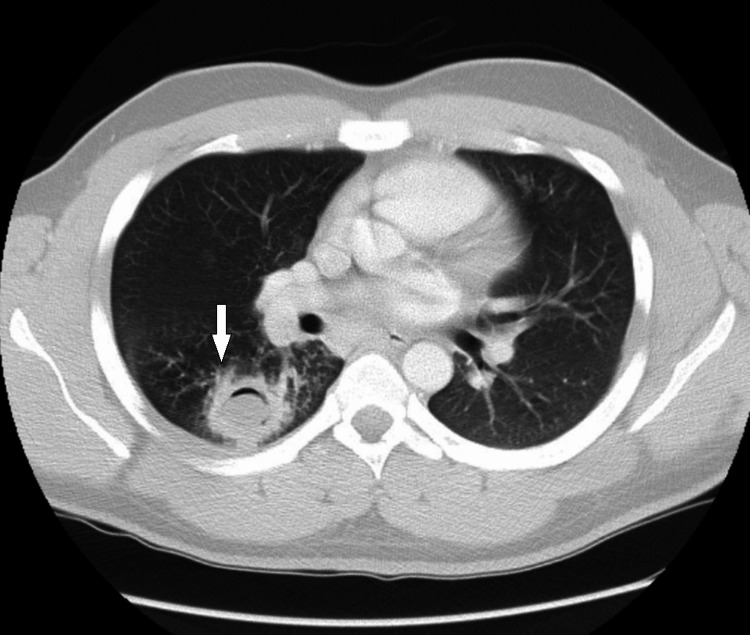
Chest CT scan showing the right lung abscess (arrow)

The PCP then ordered a contrast-enhanced chest CT scan, which was completed on the day prior to presenting to our clinic. The CT scan identified a 3.2 cm cavitary lesion in the superior segment of the right lower lobe, with irregular margins and central cavitation, interpreted by the radiologist as being “suspicious for either an inflammatory mass, neoplasm, or aspergilloma” (Figure 2).

On presentation to the clinic, the patient appeared well-nourished and in no acute distress. His vital signs in the clinic were as follows: temperature of 97.5°F, mildly elevated blood pressure at 137/92 mmHg, pulse of 89 bpm, respiratory rate of 16 breaths per minute, and an oxygen saturation of 97% on room air. On physical examination, the patient’s oral cavity showed healthy gums and teeth, with no abnormalities. Pulmonary auscultation revealed clear lung fields bilaterally, with no wheezing, rales, or rhonchi detected. Cardiac examination showed a regular rate and rhythm, with no murmurs, gallop, or rub present. The rest of the physical exam was normal

The patient was referred for emergent hospital admission for inpatient management in view of the concerning CT findings and was placed under precautionary respiratory isolation to rule out tuberculosis. Admission vital signs and physical examination findings were unchanged from the normal findings reported in the clinic. Significant admission laboratory results are summarized in Table 1.

**Table 1:** Laboratory results on admission

Parameter	Result (Normal Range)
Hemoglobin	10.8 (13.8 – 17.2 g/dL)
WBC (Absolute Count)	8.9 (3.5 - 10.5 x 10^3^ cells/μL)
Neutrophils (Absolute Count)	6.6 (1.7 – 7.0 x 10^3^ cells/μL)
Serum Albumin	2.8 (3.5 – 5.0 g/dL)
Serum Calcium	7.2 (8.5 – 10.2 mg/dL)
Ferritin	531.6 (24 – 336 ng/mL)
Serum Iron	22 (60 – 170 µg/dL)
Iron Saturation	12.5 (20% – 50%)
Procalcitonin	0.04 (Less Than 0.1 ng/mL)

Pulmonology and Infectious Disease (ID) were consulted, and additional laboratory studies were ordered. Notably, antibiotics were not ordered as the patient did not show any “toxic” signs or symptoms. Inpatient daily medical progress notes and the consultant notes emphasized the lack of fever and chills along with the normal WBC in their decision not to begin antibiotics. Additional serologic tests, including HIV, Aspergillus galactomannan, ANCA, and ACE levels, were negative. Three sputum samples for acid-fast bacilli returned negative by day 5 of admission, allowing for the removal of respiratory isolation precautions. During this time, his temperatures ranged between 97.5 and 99.6, until day 8, when he developed a maximum temperature of 100.6 F. He did not develop any leukocytosis. On admission day 8 ID started the patient on oral amoxicillin-clavulanate and sulfamethoxazole-trimethoprim. The patient's fever and hemoptysis did not improve with these antibiotics.

Cardiothoracic surgery was consulted on day 9 and recommended surgical biopsy with possible resection to make a definitive diagnosis. On day 11, cardiothoracic surgery performed a video-assisted thoracoscopy and resected the right lower lobe. The patient was started on intravenous cefazolin 1g post-operatively. Pathology of the right lower lobe was negative for malignancy, tuberculosis, fungal infection, and autoimmune disorders. Pathology reported that the cavitary mass, measuring 4.5 cm, was filled with red-brown clotted blood. Histologic examination revealed organizing pneumonia with marked pulmonary edema and congestion. Histopathology of the hilar lymph nodes revealed marked follicular lymphoid hyperplasia and anthracosis. All cultures were negative. The pathologist suggested aspiration pneumonia as the most likely etiology.

A post-surgical chest X-ray revealed a right-sided hydropneumothorax, estimated at 30%, with no mediastinal shift, despite two chest tubes having been placed in the right hemithorax. Serial chest imaging showed decreasing size of the pneumothorax, and the patient remained asymptomatic with oxygen saturation between 98 and 100% on room air. One chest tube was removed on hospital day 14, followed by the other chest tube on day 15. He was monitored and treated with chest physiotherapy for three more days due to a persistent right pneumothorax and subsequently discharged on day 18 with a small, stable pneumothorax. Discharge medications included an additional five-day oral course of amoxicillin-clavulanate (875-125 mg) and sulfamethoxazole-trimethoprim (800-160 mg).

One week post discharge, the patient was seen in the medical continuity clinic for follow-up. Chest X-ray one week after discharge showed a decrease of the residual pneumothorax and pleural effusion. By one month post-op, the effusion and pneumothorax had nearly completely resolved. Follow-up at second and third months confirmed complete resolution of both the pneumothorax and pleural effusion, with the patient remaining clinically stable throughout.

Significantly, it was only at the post-discharge clinic visit that the patient revealed a pattern of recurrent binge drinking with associated episodes of unconsciousness, which provided critical insight into the etiology of aspiration with lung abscess.

## Discussion

This case highlights the variable presentation of lung abscesses. Our patient presented only with a persistent productive cough, lacking typical signs and symptoms such as fever, chills, weight loss, or night sweats. There were no abnormalities noted on the pulmonary exam. He did not develop any leukocytosis. Symptoms of an acute lung abscess often mimic pneumonia and typically include fever, chills, cough, night sweats, dyspnea, weight loss, fatigue, and chest pain. Initially, the cough may be non-productive; however, once the abscess communicates with the bronchus, the cough becomes productive, often with purulence or hemoptysis. In chronic lung abscesses, finger clubbing may also develop [4].

Primary lung abscesses typically occur in healthy individuals and are usually isolated, as was the case with this patient, while secondary abscesses arise in patients with underlying medical conditions and often involve multiple pulmonary collections. Abscesses can be classified as "non-specific" if no specific pathogens are identified or as "putrid" when anaerobic bacteria are involved. Most abscesses are polymicrobial; commonly involving anaerobes such as Bacteroides, Prevotella, or Streptococcus species. Monomicrobial abscesses may be caused by pathogens like Staphylococcus aureus or Klebsiella pneumoniae. In patients with alcohol use disorder, Staphylococcus aureus, Klebsiella pneumoniae, and Actinomyces are frequent pathogens with poor dental hygiene also increasing this risk [2].

Lung abscesses are more common in male patients over the age of 50 with a history of alcohol abuse or seizure disorders, as these conditions may result in altered consciousness, increasing the risk of aspiration [6]. In our patient, who was under 30, the differential diagnosis initially considered tuberculosis, aspergilloma, Wegener’s granulomatosis, Goodpasture’s syndrome, sarcoidosis, and pneumoconiosis. At the time of hospital admission, aspiration pneumonia was not given much consideration. The dearth of infectious symptoms with the imaging findings of a cavity inside a mass biased the clinician's differential diagnosis away from considering aspiration pneumonia as a likely etiology. Acid-fast bacilli (AFB) and fungal sputum testing and serologic studies were all negative, thereby obligating the medical team to perform further invasive procedures to clarify the diagnosis.

Intravenous antibiotic therapy is the cornerstone of initial lung abscess management, with 80-90% of patients responding well to antimicrobial treatment Some studies quote up to 95% of patients responding adequately to IV antibiotics [7]. Standard empiric therapy typically involves beta-lactamase inhibitors such as ticarcillin-clavulanate, ampicillin-sulbactam, amoxicillin-clavulanate, or piperacillin-tazobactam. Blood or sputum culture results may occasionally require treatment with imipenem or meropenem [8]. For patients unresponsive to medical therapy, surgical intervention with lobectomy or pneumonectomy may be necessary [2]. In the mid-1980s, percutaneous transthoracic drainage emerged as a less invasive alternative for patients who do not respond to medical treatment; however, this is generally reserved for patients who are at high risk for complications after a pulmonary resection [9]. 

Our patient received oral amoxicillin-clavulanate and sulfamethoxazole-trimethoprim only starting on day 8 of hospitalization. Given his persistent cough and hemoptysis, as well as the initial radiology interpretation of the chest CT scan findings, a high index of suspicion still remained for aspergillosis or non-infectious etiologies, and surgical biopsy was deemed necessary. Post-operatively, the patient developed a persistent pneumothorax, resulting in a prolonged hospital stay and prolonged clinic follow-up for close monitoring until resolution. Intravenous antibiotics were not initiated until hospital day 11, the day of his lobectomy. The patient was discharged on hospital day 18.

The importance of a thorough clinical history in directing medical investigation and treatment cannot be overstated, as evidenced by a 1975 study by Hampton, which found that a meticulous history could lead to a diagnosis in 80% of cases [10]. In the case presented here, it was only during a post-discharge clinic visit that the patient disclosed a history of binge drinking with recurrent loss of consciousness, a key detail that was initially not shared by the patient. This omission biased the medical team’s differential diagnosis away from an aspiration-related lung abscess and led to a delay in appropriate antibiotic treatment. This lack of the complete history resulted in a diagnostic bias which then led to what might have been a preventable lobectomy, with its associated surgical risk.

Sir William Osler famously advised, "Listen to your patient; he is telling you the diagnosis" [11]. This aphorism remains a timeless reminder of the enduring importance of foundational bedside skills and patient-centered care.

## Conclusions

This case underscores the critical importance of thorough history-taking in diagnosing pulmonary lesions, particularly in young patients presenting with atypical symptoms. The patient's delayed disclosure of recurrent binge drinking and associated episodes of unconsciousness would have been pivotal in suspecting aspiration as the underlying etiology of the lung abnormality seen on the chest CT scan. Recognizing this history earlier could have facilitated timely initiation of targeted intravenous antibiotic therapy, potentially preventing the progression of the abscess and the need for invasive interventions.

The case serves as a reminder of the enduring value of fundamental clinical skills, such as comprehensive history-taking, even in an era dominated by advanced diagnostic technologies. Clinicians must remain vigilant in practicing patient-centered care, fostering trust, and encouraging open communication to uncover critical historical details. These efforts are essential for accurate diagnosis, effective management, and, ultimately, improved clinical outcomes for every patient.
